# Global research activity on antimicrobial resistance in food-producing animals

**DOI:** 10.1186/s13690-021-00572-w

**Published:** 2021-04-13

**Authors:** Waleed M. Sweileh

**Affiliations:** Department of Physiology, Pharmacology/Toxicology, Division of Biomedical Sciences, College of Medicine and Health Sciences, An-Najah National University, Nablus, Palestine

**Keywords:** Food-producing animals, Antimicrobial resistance, Bibliometric analysis, Scopus

## Abstract

**Background:**

Antimicrobial resistance (AMR) is a global challenge that requires a “One Health” approach to achieve better public health outcomes for people, animals, and the environment. Numerous bibliometric studies were published on AMR in humans. However, none was published in food-producing animals. The current study aimed at assessing and analyzing scientific publications on AMR in food-producing animals.

**Method:**

A validated search query was developed and entered in Scopus advanced search function to retrieve and quantitatively analyze relevant documents. Bibliometric indicators and mapping were presented. The study period was from 2000 to 2019.

**Results:**

The search query retrieved 2852 documents. During the period from 2015 to 2019, approximately 48% of the retrieved documents were published. The article about the discovery of plasmid-mediated colistin resistance in pigs received the highest number of citations (*n* = 1970). The *Journal of Food Protection* (*n* = 123; 4.3%) ranked first in the number of publications while the *Applied and Environmental Microbiology* journal ranked first in the number of citations per document. The USA led with 576 (20.2%) documents followed by China (*n* = 375; 13.1%). When the number of publications was standardized by income and population size, India (*n* = 51.5) ranked first followed by China (*n* = 38.3) and Brazil (*n* = 13.4). The growth of publications from China exceeded that of the USA in the last 3 years of the study period. Research collaboration in this field was inadequate. Mapping author keywords showed that *E. coli*, Salmonella, poultry, Campylobacter, chicken, cattle, and resistant genes were most frequent. The retrieved documents existed in five research themes. The largest research theme was about AMR in Salmonella in food-producing animals. The most recent research theme was about the dissemination and molecular transfer of AMR genes into the environment and among different bacterial strains.

**Conclusion:**

Hot spots of research on AMR in food-producing animals match the world regions of reported hot spots of AMR in animals. Research collaboration in this field is of great importance, especially with low- and middle-income countries. Data on AMR need to be collected nationally and internationally to implement the “One Health” approach in the fight against AMR.

**Supplementary Information:**

The online version contains supplementary material available at 10.1186/s13690-021-00572-w.

## Background

Food-producing animals may get exposed to antimicrobials upon the use of antimicrobials to act as animal growth promoters or as a prophylactic or therapeutic measure [1]. Antimicrobials that are used in animals and humans are closely related [2]. Therefore, antimicrobial resistance (AMR) in animals is of concern to human health [3]. Recent literature has described AMR in farm animals as a huge problem that has not been given adequate attention [4]. Antimicrobial resistance in animals poses a double problem by causing untreatable infections in humans and animals. A study in low- and middle-income countries about AMR indicated that farm animals in India, northeast China, Kenya, Uruguay, and Brazil are becoming more resistant to common antimicrobial drugs [5]. The emergence of plasmid-mediated resistance against colistin in pigs in China [6] has increased the concern about the use of antimicrobials in food-producing animals. The mobilized colistin resistance-1 gene (MCR-1) was identified in 2014 in China but soon became present in many countries [7].

The misuse and/or overuse of antimicrobial agents in humans and animals played an important role in developing AMR [8]. The exposure of food-producing animals to antimicrobials is expected to increase globally given the increased human demand for food especially in countries with a large number of populations [9]. The same study indicated that global consumption of antimicrobials in food animal production was estimated at 63,151 (±1560) tons in 2010 and is projected to rise by 67%, to 105,596 (±3605) tons, by 2030.

The problem of AMR requires a “One Health” approach in which the health of humans is considered closely connected to the health of the animals and the environment [10]. The Food and Agriculture Organization of the United Nations (FAO), the World Organization for Animal Health (OIE), and the WHO are collaborating to develop evidence-based guidelines for use of medically important antimicrobials in food-producing animals to minimize health threats at the human-animal-ecosystems interface. Limited direct evidence was found between the reduction of antibiotic use in food animals and AMR in humans suggesting complex and long-term pathways rather than a direct and simple causal pathway between AMR in food animals and human health [11]. The direct contact of humans with animals or the consumption of contaminated foods of animal origin, or consumption of contaminated water and vegetables remain the obvious link between animals and human health [12]. Antibiotics administered to animals are excreted either as the parent compound or as metabolites in the urine and feces [13] and persist in the environment for adequate time to select for resistant bacteria in the soil even at a lower concentration. Furthermore, horizontal transfer of antimicrobial-resistant genes in the environment further disseminates AMR [14].

It was argued that AMR might cause millions of human deaths in the future due to infections caused by resistant microbes [15] and that is why AMR is considered by the World Health Organization (WHO) as one of the top ten global health threats that need urgent action [16]. Because of this, there is a pressing need for monitoring research activity on AMR in foods and food products of animal origin. This is important for national and international policymakers to draw guidelines on the use of antimicrobials in animal feed. Furthermore, assessing and monitoring research activity on AMR in food-producing animals will help understand the emerging AMR for some of the last-resort antibiotics such as colistin. Assessing the research activity on AMR in animal-producing food is one method to track emerging resistant pathogens that are common between animals and humans. Therefore, the current study aimed to give academics, scientists, researchers, and health experts interested in global health security a detailed analysis of literature published on AMR in food-producing animals. In specific, volume and annual growth of publications, citation analysis, research themes, frequently encountered author keywords, active key players, and international research collaboration was investigated. Such detailed analysis is the first of its type and will be the baseline for future comparison on various aspects of AMR in food-producing animals. The current study is a quantitative bibliometric description of the literature on AMR in food-producing animals and not a systematic or scoping review. Several bibliometric studies on various medical subjects have been published [17, 18].

## Materials and methods

### Database

In the current study, the literature on AMR in food-producing animals published in peer-reviewed journals indexed in SciVerse Scopus was retrieved. The selection of SciVerse Scopus was based on the advantages it has over other databases such as Web of Science and PubMed [19]. Scopus offers an advanced search function, which allows researchers to build comprehensive search queries using different functions and different Boolean operators. In the current study, the advanced search function was used.

### Search query

The search query was developed based on terms related to AMR in food-producing animals listed in the OIE report [20]. The terms used included names of all antibiotics and names of all food-producing animals listed in the OIE report. In addition to the specific terms listed in the OIE report, general phrases related to AMR were also included in the search query. The details of the search query and keywords used were shown in supplementary material 1. The search query was developed and finalized after several trials using different search scenarios to obtain the maximum accurate results. The study period was defined from January 01, 2000, to December 31, 2019. The search query included journal publications while grey literature was not included because grey literature does not represent research findings. No language restriction was imposed on the retrieved literature.

### Validation of the search strategy

The finalized search query was validated for the absence of false-positive results by ensuring that the top 200 cited documents were relevant to the search topic. The author asked two colleagues who are experts in microbiology to check for false-positive results by reading the title and abstracts of the top-cited documents sent to them as an endnote file. The search query was considered final when the experts confirmed the absence of any false-positive results. To ensure the absence of false-negative, the correlation test between what has been retrieved by the search query and the actual findings for the top ten active researchers was implemented. The result of the correlation test was significant (*p* < 0.01) and strong (*r* = 0.96) indicative of the validity of the search query. This validation approach was implemented in previously published bibliometric studies [21].

### Data export

The retrieved data in Scopus were exported to Microsoft Excel using “analyze” and “export” functions consequently. The exported data included the annual number of publications, type of documents, subject areas, journal names, countries, institutions, authors, funding agencies, citations, terms in titles/abstracts, and indexed keywords.

### Data analysis and bibliometric indicators

The annual number of publications was presented as a line graph using the Statistical Package for Social Sciences (SPSS version 21). Active journals in publishing documents on AMR in food-producing animals were presented as top ten list. The number of publications for each country was standardized by the Gross Domestic Product (GDP) per capita [22]. The international research collaboration was assessed by network visualization. In the map, the thickness of the connecting lines, as well as the distance between countries, is proportional to the strength of research collaboration. Countries located far away from the center of the map and have thin connecting lines are the ones with inadequate international research collaboration. The research themes in the retrieved literature were found by mapping the most frequent terms in titles/abstracts. A group of terms with similar colors represents a cluster, which represents a research theme. Overlay visualization was used to determine the time sequence and research trend in the retrieved literature.

## Results

### Types of documents and subject areas

The search query found 2852 documents; mostly as research articles (*n* = 2611; 91.5%) and review articles (*n* = 137; 4.8%). The remaining types of documents were letters, editorials, notes, and conference papers. The language in the documents was mainly English (*n* = 2703; 94.8%) followed by Chinese (*n* = 51; 1.8%), and Portuguese (*n* = 26; 0.9%). Other remaining languages included Germany, Spanish, Turkish, French, Korean, Japanese, and Dutch. In total, 1038 (36.4%) documents were published as open access. The retrieved documents were published in journals categorized in different subject areas, mainly in immunology/microbiology (*n* = 1164; 40.8%), followed by agriculture and biological sciences (*n* = 1104; 38.7%), medicine (*n* = 775; 27.2%), veterinary (*n* = 738; 25.9%), Biochemistry/genetics/molecular biology (*n* = 443; 15.5%), environmental sciences (*n* = 406; 14.2%), pharmacology/toxicology (*n* = 234; 8.2%), and multidisciplinary (*n* = 189; 6.6%).

### Annual growth of publications and citation analysis

**Fig. 1:**
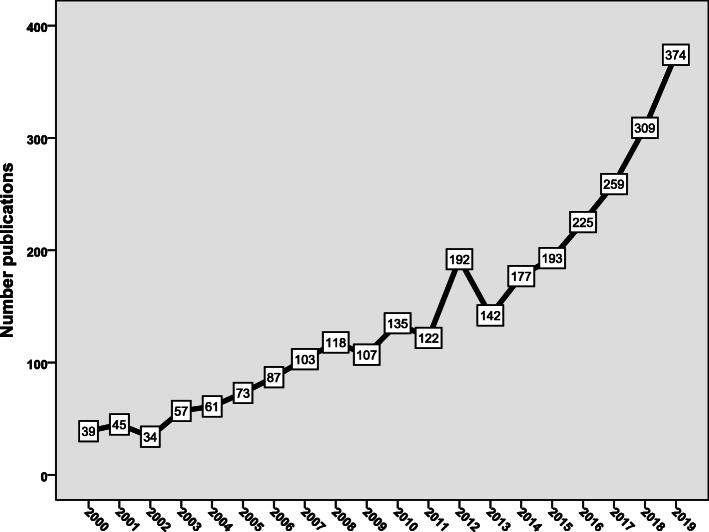
Annual growth of publications on antimicrobial resistance in food-producing animals (2000–2019)

Figure 1 shows the annual growth of publications during the study period. Approximately half of the retrieved documents (*n* = 1360; 47.7%) were published in the last 5 years (2015–2019) of the study period. The retrieved documents received 63,443 citations, an average of 22.4 citations per document. The top-cited document in this field was published in *The Lancet Infectious Diseases* in 2016 and received 1754 citations within less than 4 years, an average of approximately 439 citations per year [6]. The top-cited document discussed the discovery of a plasmid-mediated gene for colistin resistance. The discovery was made in pigs in China.

### Top ten active journals publishing the retrieved literature

Table 1 shows the list of the top ten active journals in publishing the retrieved documents. The top ten active journals published 994 (28.3%) documents. The *Journal of Food Protection* (*n* = 123; 4.3%) was the most productive in this field followed by the *Foodborne Pathogens and Disease* (*n* = 109; 3.8%) and the *Applied and Environmental Microbiology* (*n* = 87; 3.1%). Despite the *Journal of Food Protection* ranked first in the number of publications*,* documents published in the *Applied and Environmental Microbiology* received the highest number of citations per document followed by those published in the *Foodborne Pathogens and Disease* journal.

**Table 1:** Top ten active journals in publishing documents on antimicrobial resistance in food-producing animals (2000–2019)

Rank	Country	Frequency	% *N* = 2852	Citations per document	Country
1	*Journal of Food Protection*	123	4.3	19.7	USA
2	*Foodborne Pathogens and Disease*	109	3.8	21.2	USA
3	*Applied and Environmental Microbiology*	87	3.1	67.2	USA
4	*Frontiers in Microbiology*	73	2.6	16.4	Switzerland
5	*International Journal of Food Microbiology*	71	2.5	41.4	Netherlands
6	*Veterinary Microbiology*	69	2.4	29.1	Netherlands
7	*Microbial Drug Resistance*	66	2.3	20.4	USA
8	*PLOS ONE*	54	1.9	16.1	USA
9	*Journal of Antimicrobial Chemotherapy*	52	1.8	59.9	UK
10	*Food Control*	42	1.5	21.1	Netherlands

### Active countries

**Fig. 2:**
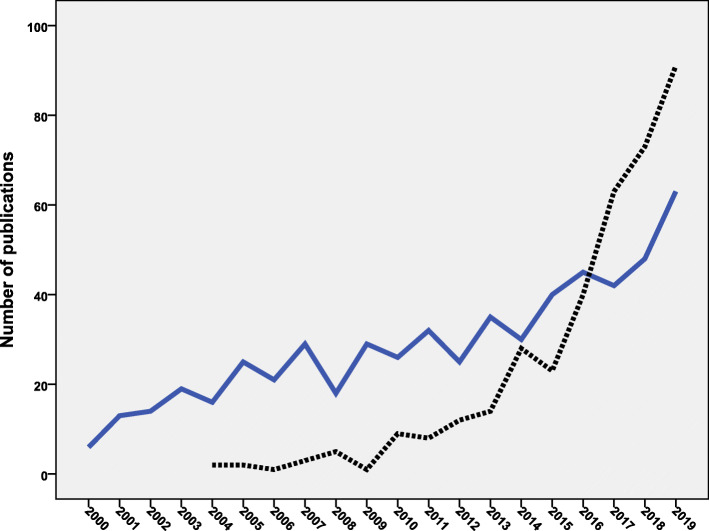
Comparison of annual growth of publications from the USA (blue line) and China (black dotted line) on antimicrobial resistance in food-producing animals (2000–2019)

The top ten active countries were listed in Table 2. The active countries contributed to 1967 (69.0%) of the retrieved documents. The list of active countries included two countries in Northern America, one in South America, three in Europe, and four in Asia. The USA led with 576 (20.2%) documents followed by China (*n* = 375; 13.1%) and Canada (*n* = 189; 6.6%). When the number of publications was standardized by GDP (nominal) per capita, India (*n* = 51.5) ranked first followed by China (*n* = 38.3) and Brazil (*n* = 13.4). Figure 2 compares the growth of publications from the USA and China. The annual growth of publications from the USA started earlier. However, the annual number of publications from China noticeably exceeded those from the USA after 2016. Analysis of the retrieved literature based on world regions showed that the European region had the highest contribution (*n* = 1009; 35.4%) followed by the region of the Americas (*n* = 902; 31.6%). The contribution of each of the six world regions was shown in Table 3.

**Table 2:** Top ten active countries in publishing documents on antimicrobial resistance in food-producing animals (2000–2019)

Rank	Country	Frequency	% *N* = 2852	Number of publications/ GDP (nominal) per capita (10^−3^)^a^
1	United States	576	20.2	10.9
2	China	375	13.1	46.2
3	Canada	189	6.6	4.5
4	United Kingdom	155	5.4	4.8
5	Spain	123	4.3	5.2
6	Japan	109	3.8	3.7
7	Germany	110	3.9	2.9
8	Brazil	119	4.2	15.4
9	South Korea	108	3.8	4.1
10	India	103	3.6	54.0

^a^Number of publications/GDP (nominal) per capita for a particular country was calculated by dividing the number of publications by the GDP (nominal) per capita for that country. Data on GDP (nominal) per capita was obtained from the World Bank data

**Table 3:** Number of publications on AMR in food-producing animals by world region (2000–2019)

Region^a^	Number of countries in the region	Number of publications	% (*N* = 2852)
African region	47	197	6.9
The region of the Americas	35	902	31.6
European region	54	1009	35.4
The South-East Asian region	11	202	7.1
The Eastern Mediterranean region	21	240	8.4
Western Pacific region	27	620	21.7

^a^Region classification was based on the World Health Organization data

### International research collaboration

**Fig. 3:**
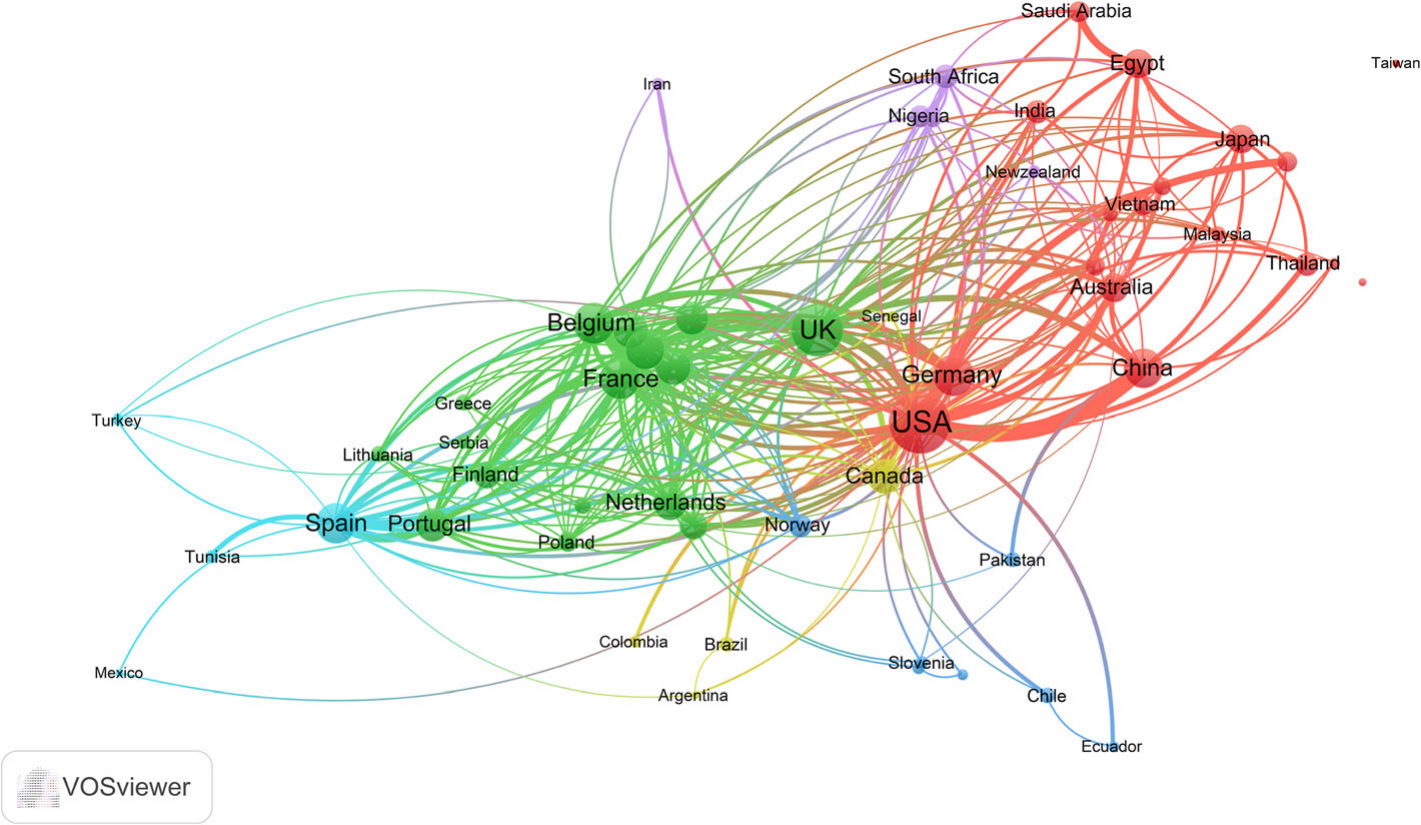
Network visualization map of international research collaboration for countries with a minimum contribution of 10 documents on antimicrobial resistance in food-producing animals (2000–2019)

Countries with a minimum contribution of 50 documents were presented as a network visualization map (Fig. 3) to assess international research collaboration. The map showed that the majority of countries were located far away from the center and had thin limited connection lines with other countries indicative of inadequate international research collaboration. The map showed that six European countries (Switzerland, Italy, Belgium, Denmark, France, and Sweden) had a close research connection and collaboration. The map showed that the strongest research collaboration in this field was between the USA and China with a link strength of 49 followed by that between the USA and Canada (link strength = 30).

### Most encountered author keywords

**Fig. 4:**
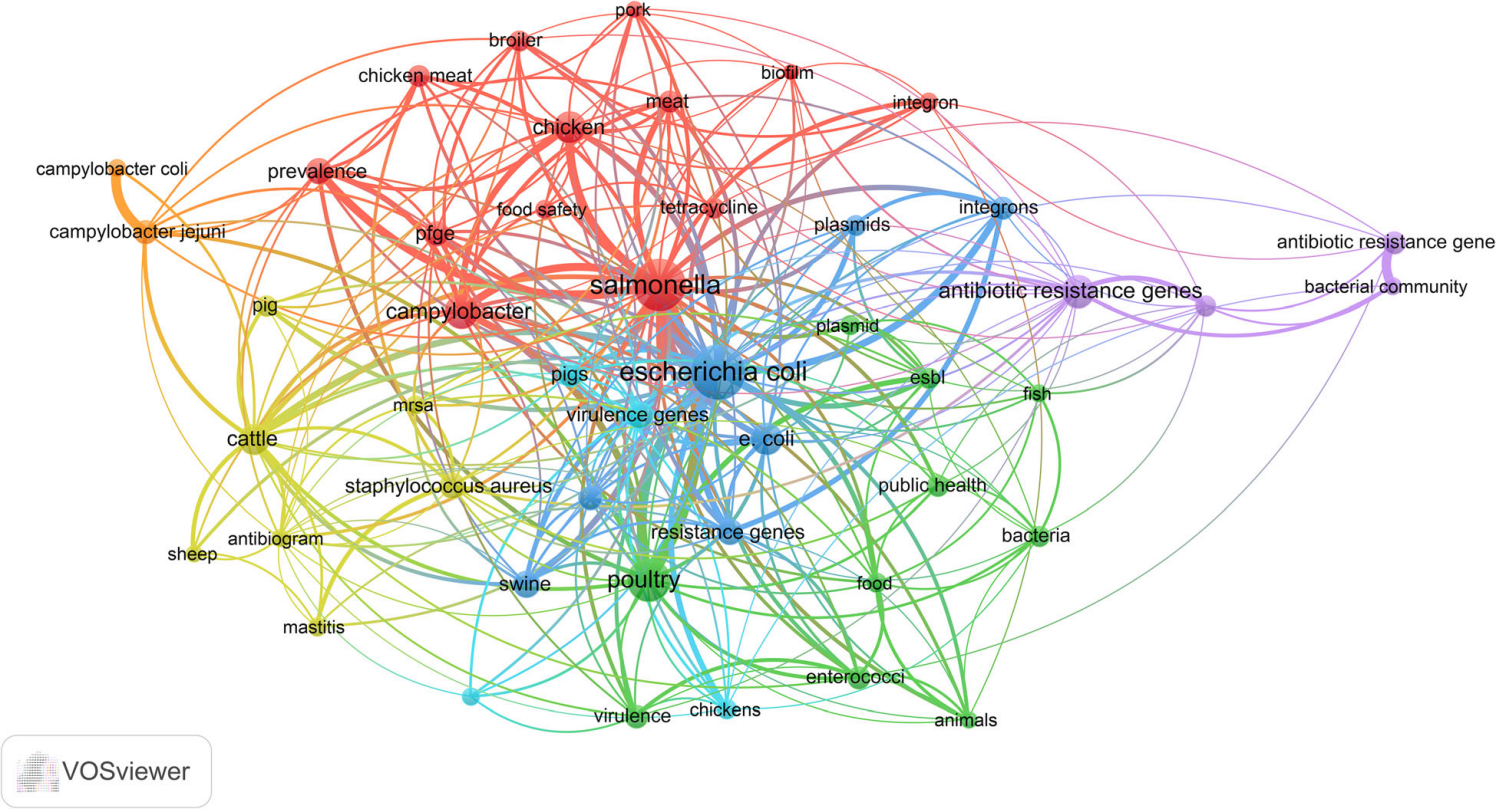
Network visualization map of frequent author keywords on antimicrobial resistance in food-producing animals (2000–2019)

**Fig. 5:**
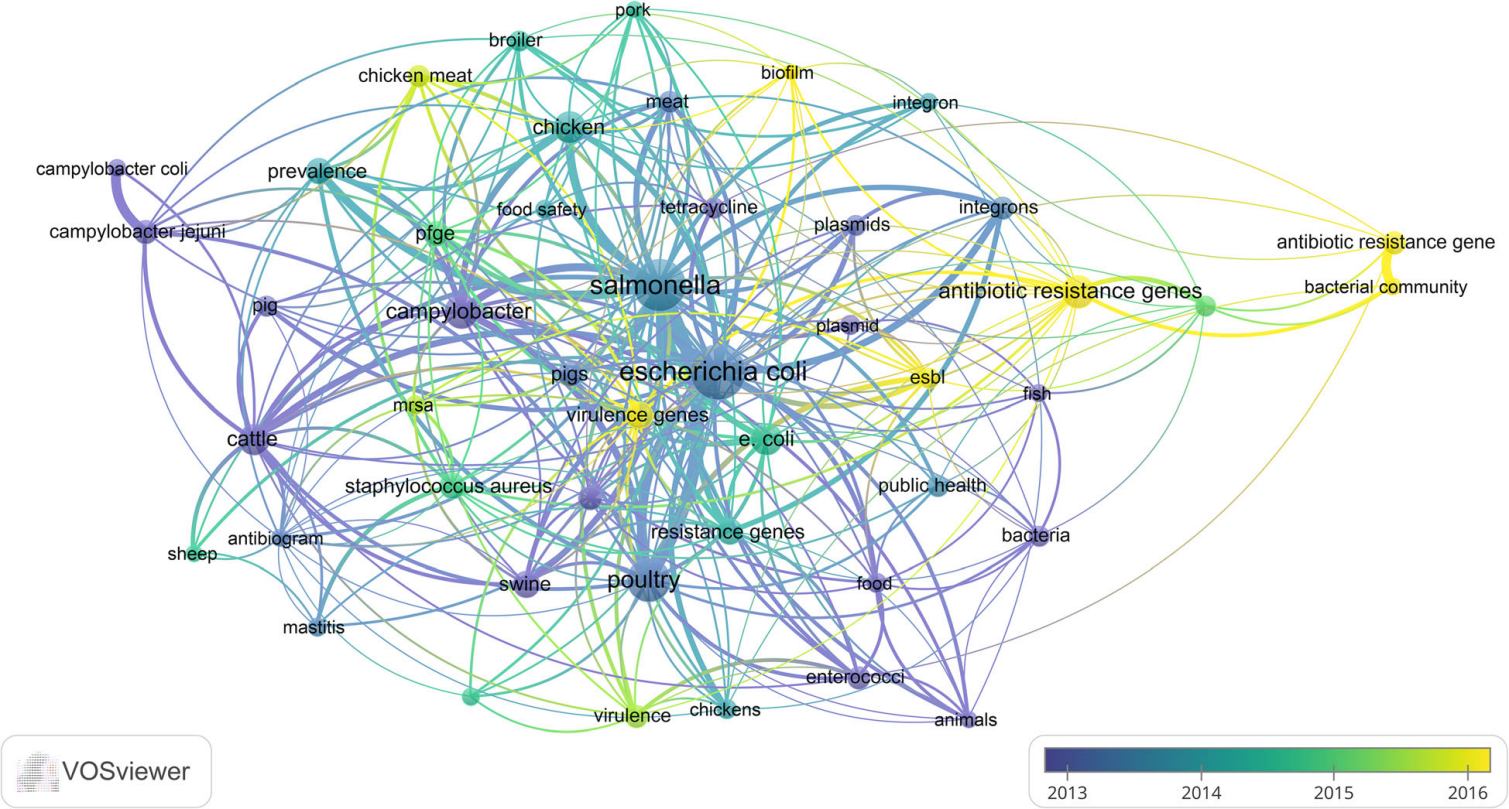
Overlay visualization map of frequent author keywords showing the time sequence of frequent terms on antimicrobial resistance in food-producing animals (2000–2019). Yellow terms represent the most recent literature

Mapping author keywords showed that *E. coli*, *Salmonella*, poultry, *Campylobacter*, chicken, cattle, and resistant genes were most frequent (Fig. 4). Overlay visualization of the same author keywords to map the time sequence of these author keywords showed that resistant genes, ESBL, biofilm, and virulence genes were introduced after 2016 while other keywords present in the map were introduced before 2016 (Fig. 5).

### Research themes

**Fig. 6:**
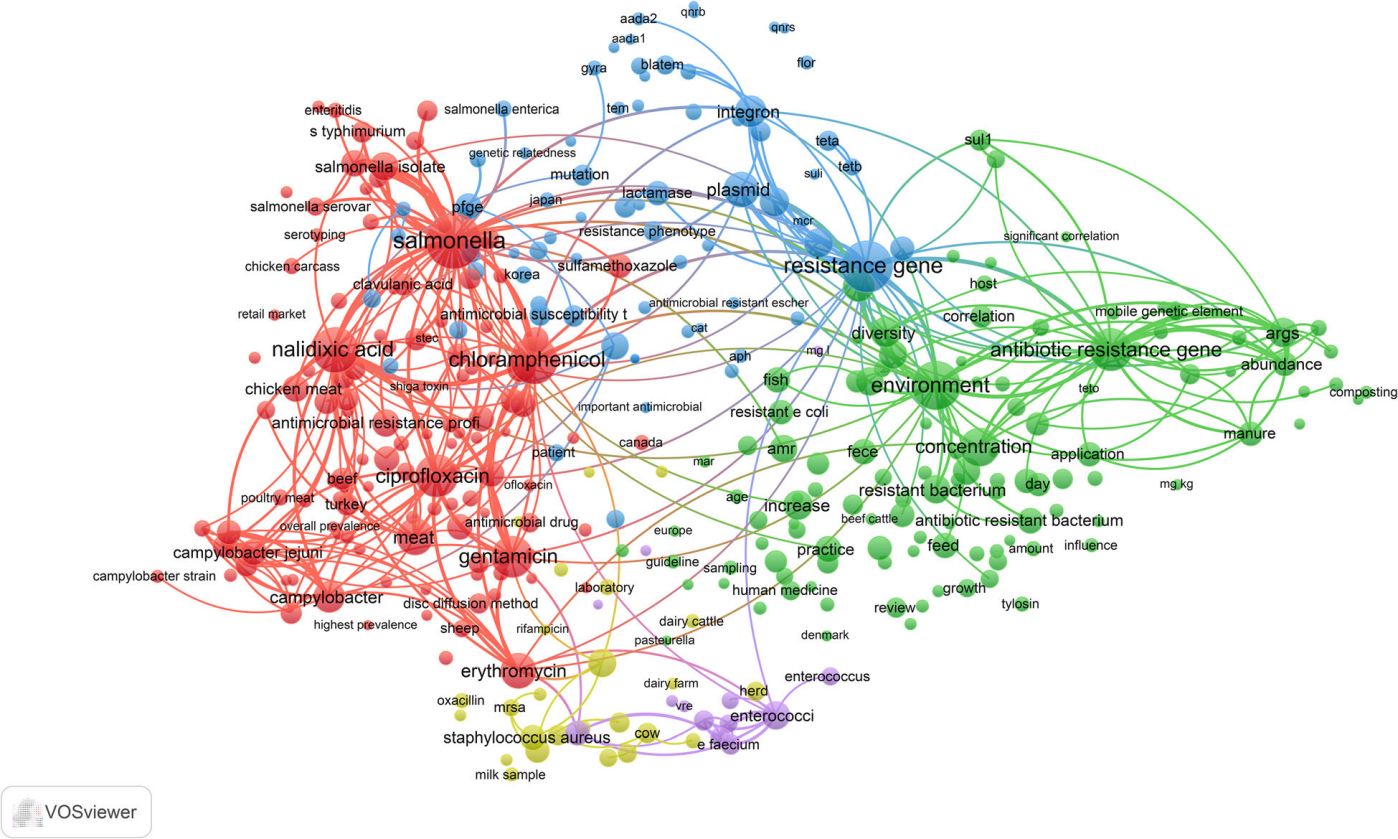
Network visualization map of the most frequent terms in titles/abstracts of literature on antimicrobial resistance in food-producing animals (2000–2019)

**Fig. 7:**
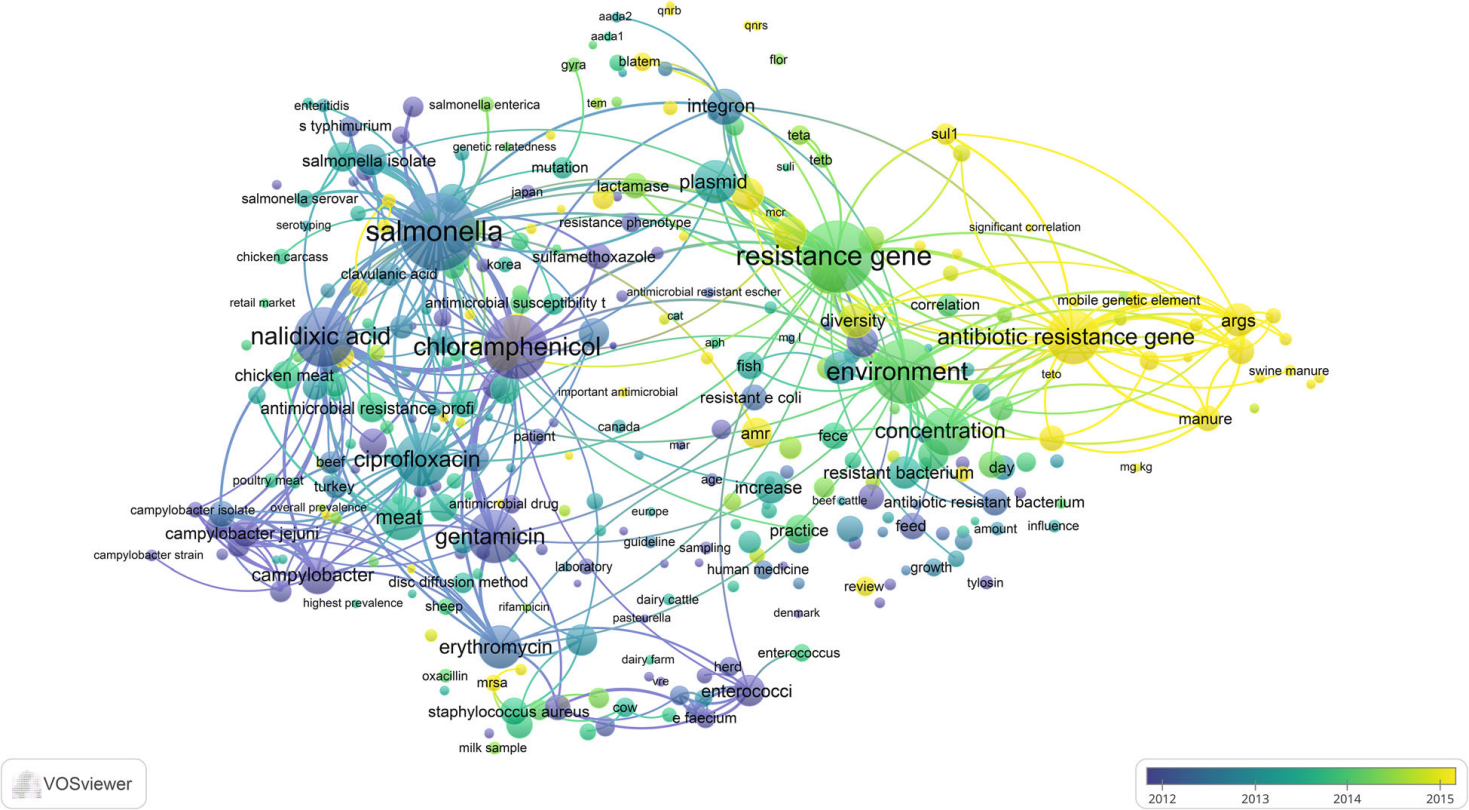
Overlay visualization map of frequent terms in titles/abstracts of the retrieved literature showing the time sequence of research themes on antimicrobial resistance in food-producing animals (2000–2019). The yellow cluster represents the most recent literature

Mapping terms in the titles and abstracts of the retrieved literature yielded five clusters repressing five major research themes in this field (Fig. 6). The first cluster (green) represents a research theme on the dissemination of antimicrobial-resistant genes from animals to the environment. The second cluster (blue) is a research theme focusing on molecular development and transfer of antimicrobial-resistant genes from one bacterial strain to another through plasmids. This research theme seems to be associated with studies in China. The third cluster (red) is the largest theme and discussed antimicrobial resistance in *Salmonella* with special reference to chloramphenicol, nalidixic acid, ciprofloxacin, and gentamicin. The fourth cluster (light purple) is a research theme that discussed *Enterococcus* and vancomycin resistance. The fifth cluster (dark yellow) is a research theme that mainly discussed MRSA and penicillin. The dissemination and molecular mechanisms of antimicrobial resistance genes represent the most recent research themes in the field as shown in the overlay visualization map with yellow color being most recent (Fig. 7).

### Funding and active institutions/organizations

Analysis indicated that 1162 (40.7%) documents received funding. The major funding agency in this field was the “*National Natural Science Foundation of China*” (*n* = 150; 5.3%). The “United States Department of Agriculture” (*n* = 108; 3.8%) was the most active institution followed by the “Technical University of Denmark” (*n* = 62; 2.2%), “University of Guelph” (Canada) (*n* = 66; 2.3%), “Food and Drug Administration” (*n* = 59; 2.1%), “Chinese Academy of Sciences” (*n* = 62; 2.2%), “Public Health Agency of Canada” (*n* = 58; 2.0%), and “Agriculture and Agri-Food Canada” (*n* = 45; 1.6%). The active institutions included academic, research, and governmental institutions.

## Discussion

The current study was a descriptive study on global research output on AMR in food-producing animals. In May 2015, the World Health Assembly adopted the global and national action plan on AMR. The plan was supported by a manual developed by WHO, in collaboration with the FAO and the OIE to help countries in preparing and developing their national action plans on AMR [23]. Therefore, countries across the world are actively involved in implementing national action plans to fight AMR in a “One Health” approach. The noticeable growth of publications observed in the last few years of the study period reflects national and international commitment to fight AMR in a “One Health” approach.

The current study showed that the top-cited document was about a plasmid-mediated resistance gene (MCR-1) isolated from pigs in China [6]. This publication is considered important in the field since it explained the rapid dissemination of resistance among Gram-negative bacteria for colistin, a cyclic cationic peptide antibiotic used as a last-resort for gram-negative infections caused by *Acinetobacter baumannii* or *Pseudomonas aeruginosa* [24]. The bacterial gene responsible for colistin resistance is capable of horizontal transfer, which is a major reason for the fast spread of colistin resistance [25]. Colistin is used in animals to treat infections caused by *Enterobacteriaceae* and as a growth promoter. The unregulated use of colistin in animals played a major role in the development of resistant genes in bacteria [26]. The MCR-1 gene was identified in *Escherichia coli*, *Klebsiella*, *Salmonella*, *Shigella*, and *Enterobacter* in humans and animals [27].

The current study indicated that China, India, Japan, and South Korea were among the top active countries in the field. A recently published research paper indicated that India and China are hot spots of AMR in animals and that Brazil and Kenya are newly emerging hot spots [5]. The authors of the paper believed that the low prices and unregulated use of antimicrobial agents in food-producing animals are the reasons behind the hot spots in India and China. A study expected an increase in global consumption of antimicrobials by 67% by 2030 and that consumption in Asia is projected to increase to 51,851 tons [9]. The authors of the paper reported that in 2010 China (23%), the United States (13%), Brazil (9%), India (3%), and Germany (3%) had the largest share of global use while in 2030 China (30%), the United States (10%), Brazil (8%), India (4%), and Mexico (2%) will have the largest share of antimicrobial use in animal food.

The AMR is a global problem and therefore international research collaboration in this field is important. The current study showed inadequate global research collaboration in this field. The use and regulations of antibiotics in animals differ from one region to another and therefore research collaboration will help in knowledge transfer and implementing policies to avoid future problems. Furthermore, researchers and academics in developing countries need to collaborate and build research bridges with scientists in hot spots for AMR in animals to transfer the knowledge to their countries.

The current study indicated that resistant pathogens in food-producing animals included both gram-positive and negative ones. The most common drug-resistant foodborne bacteria of relevance to human health were *Salmonella*, *Campylobacter*, and *E. coli* [28]. *Salmonella* bacteria are prevalent in food animals such as poultry, pigs, and cattle. Salmonellosis affects humans through the consumption of contaminated food of animal origin (mainly eggs, meat, poultry, and milk). A recent study in Brazil investigated the occurrence of resistance in *Salmonella* spp., isolated from products and raw material of animal origin (swine and poultry) to antimicrobials found that 51 (38%) out of 134 isolates were resistant to at least one of the eight antibiotics used, and 28 (55%) of resistant isolates were multi-resistant [29]. A recently published systematic review on the prevalence of antibiotic resistance in *E. coli* strains simultaneously isolated from humans, animals, food, and the environment indicated that colistin had the lowest prevalence and amoxicillin the highest in isolated human *E. coli* strains [30]. The systematic review also indicated that the prevalence of Extended-Spectrum Beta-Lactamase (ESBL)-producing *E. coli* was highest in animals compared to human or environmental/food isolates. A study of the global and regional burden of 22 foodborne diseases indicated that the leading cause of the foodborne illness was norovirus followed by campylobacter. The diarrheal and invasive infections caused by non-typhoidal *Salmonella enterica* infections caused the largest burden of disease. The authors found that the burden of foodborne illness was highest in WHO’s African region [31].

Enterococci such as *Enterococcus faecalis* and *Enterococcus faecium* were reported in the current study and had been reported to have intrinsic and acquired resistance to a wide range of antibiotics including vancomycin [32]. Currently, vancomycin-resistant enterococci (VRE) is a challenge in clinical settings [33]. The emergence of VREs in food-producing animals was attributed to the widespread use of avoparcin in the 1990s in Europe for growth-promotion in animals [34]. In North America, the emergence of VRE in animals was not seen until 2008 and was attributed to the extensive use of vancomycin in clinical settings [35]. *Staphylococcus aureus* is another gram-positive opportunistic pathogen in animals and harbors several AMR genes [36].

The current study listed B-lactams, aminoglycosides, and Quinolones/fluoroquinolones as the most commonly encountered antibiotics drug classes in the retrieved literature. These drug classes are important therapeutic choices in human health. These drugs were listed as critically important drugs in human medicine [37]. The misuse/overuse of these drug classes threatens the efficacy and safety of antibiotics in clinical use and governmental action is needed. The fast development of chloramphenicol resistance upon use in animals led the FDA to ban the use of chloramphenicol in food-producing animals [38]. The limited number of new antibiotics and the rapid emergence of AMR require re-thinking of antibiotic use in animal producing animals by preventing the use of antibiotics as growth promotors, restricting the use of antibiotics to prescriptions by veterinary specialists, and implementing antibiotic resistance monitoring program. There are different positions on the ban of antibiotics in animals. The debate is due to the negative economic consequences and the inadequate protein production for human nutrition.

### Limitation

The current study is the first study to analyze AMR literature in food-producing animals hoping to benefit researchers and health policymakers. However, the current study has a few limitations. First, the search query might have missed some documents due to the wide range of animals, birds, and aquatic species as well as the wide range of antibiotics used for food production. Second, the hot spots of the world such as China, Brazil, and India have unindexed journals with publications in the field and, therefore, were missed. Third, the current study focused on scientific literature. However, there are many national and international reports on this field published as governmental reports. Such reports were not included.

## Conclusion

There has been great concern about the AMR in the last two decades. The “One Health” platform requires a comprehensive approach for investigating AMR in humans, animals, and the environment. Numerous bibliometric studies were published on AMR in humans [39, 40]; however, none was carried out on food-producing animals. The current study showed a noticeable growth of publications on AMR in food-producing animals in the last 5 years. The discovery of plasmid-mediated colistin resistance received the highest citations indicative of the importance and seriousness of this issue. Countries designated as hot spots of AMR in animals ranked top in research and number of publications. Critically important antimicrobials such as B-lactams were reported in AMR literature in animals. Pathogens responsible for human gastrointestinal infections such as *E. coli* and *Salmonella* were encountered. Research in the field of AMR in food-producing animals was characterized by limited international research collaboration. National and international action plans to “improve” the use of antimicrobials in animals are of paramount importance.

## Supplementary Information

**Additional file 1: Appendix 1.** Search query and keywords used to retrieve documents on antimicrobial resistance associated with food-producing animals.

## Data Availability

All data presented in this manuscript are available on Scopus database using the search query listed in the methodology section.

## Abbreviations

AMR: Antimicrobial resistance
WHO: World Health Organization
USA: United States of America
UK: United Kingdom
