# Supplementary material for: Global research activity on antimicrobial resistance in food-producing animals
Source: Arch Public Health. 2021 Apr 13;79:49. doi: 10.1186/s13690-021-00572-w (PMC8045364; doi:10.1186/s13690-021-00572-w)
Supplement: Supplementary file 1 — Additional file 1: Appendix 1. Search query and keywords used to retrieve documents on antimicrobial resistance associated with food-producing animals. [file 13690_2021_572_MOESM1_ESM.docx]

**Bibliometric analysis of global research activity on antimicrobial resistance in food - producing animals (2000 – 2019)**

**Supplementary material 1**: Search query and keywords used to retrieve documents on antimicrobial resistance associated with food-producing animals.

| **Step** | **Boolean Operator** | **Search query** | **Number of documents** |
| --- | --- | --- | --- |
| **1** |  | title ( "antimicrobial resist*" or "antibiotic resistance" or "antibiotic resistant" or "drug* resist*" or "multi-drug resist*" or "multi-drug resist*" or "multiple-drug resist*" or "multiple drug* resist*" ) and title ( meat or chicken or fish or "food animal*" or "farm animal*" or "domestic animal*" or "livestock animal*" or "poultry" or "pig" or "cattle" or "sheep" or "goat" or "swine" or "animal feed" or "farm animal" or "cow*" or sheep or broiler* or rabbits or bees or cervidae or aquaculture or "aquafarming" or amphibians or crustaceans or mollusc or reptiles or pork ) and not title ( "cell line" or pet* or insulin or tolerance ) | **2465** |
| **2** | **OR** | title ( resist* ) and title ( erythromycin or colistin or polymyxin* or *quinolone* or "*tetracycline" or vancomycin or aminoglycoside or gentam*cin or antibacterial or penicillin or sulfonamides or macrolide or streptogramin* or kanamycin or neomycin or doxycycline or spiramycin or fosfomycin or chloramphenciol or bacitracin or cephalosprin or ceftriaxone or ampicillin or amoxicillin or antibiotic* or antimicrobial* or "*quinolone*" or lincosamides or polymyxin* or clindamycin or levoflox* or cefalexin or cefazoline or cefepime or cefoperaz* or nitrofuran or meropenem or rifampicin or tobramycin or tigecycline or b-lactam or kanamycin or apramycin or amikacin or spectinomycin or tobramycin or trimethoprim or sulfamethox* or *trimoxaz* or amoxyc* or amoxic* or ofloxacin or *floxacin or ciprofloxacin or nitrofuran or glycopeptide* ) and title ( meat or chicken or fish or "food animal*" or "farm animal*" or "domestic animal*" or "livestock animal*" or "poultry" or "pig" or "cattle" or "sheep" or "goat" or swine or "animal feed" or "farm animal" or animal or "cow*" or sheep or broiler* or rabbits or bees or cervidae or aquaculture or "aquafarming" or amphibians or crustaceans or mollusc or reptiles or pork ) and title-abs ( "antimicrobial resist*" or "antibiotic resistance" or "antibiotic resistant" or "drug* resist*" or "multi-drug resist*" or "multi-drug resist*" or "multiple-drug resist*" or "multiple drug* resist*" ) and not title ( "cell line" or pet* or insulin or tolerance ) | **3074** |
| **3** | **OR** | title ( "antimicrobial resist*" or "antibiotic resist*" or "drug* resist*" or "multi-drug resist*" or "multi-drug resist*" or "multiple-drug resist*" or "multiple drug* resist*" ) and title ( animal ) and title-abs ( meat or chicken or fish or "food animal*" or "farm animal*" or "domestic animal*" or "livestock animal*" or "poultry" or "pig" or "cattle" or "sheep" or "goat" or swine or "animal feed" or "farm animal" or animal or "cow*" or sheep or broiler* or rabbits or bees or cervidae or aquaculture or "aquafarming" or amphibians or crustaceans or mollusc or reptiles or pork ) and title-abs ( "resist*" ) and not title ( "cell line" or pet* or insulin or tolerance ) | **710** |
| **4** | **Combine** | **#1 OR #2 OR #3 = 3433** | **3433** |
| **5** | **AND** | Limit to journal articles and exclude errata documents | **3347** |
| **7** | **AND** | **Limit to 2000 - 2019** | **2852** |
